# ToF-SIMS spectral data analysis of *Paenibacillus sp.* 300A biofilms and planktonic cells

**DOI:** 10.1016/j.dib.2025.111763

**Published:** 2025-06-09

**Authors:** Gabriel D. Parker, Andrew Plymale, Luke Hanley, Xiao-Ying Yu

**Affiliations:** a Department of Chemistry, University of Illinois Chicago, Chicago, IL 60607, United States; b Materials Science and Technology Division, Oak Ridge National Laboratory, Oak Ridge, TN 37380, United States; c Energy and Environment Directorate, Pacific Northwest National Laboratory, Richland, WA 99352, United States

**Keywords:** Time-of-flight secondary ion mass spectrometry, Paenibacillus, Biofilms, Planktonic cells, Metabolites, Fatty acids

## Abstract

Analysis of bacterial biofilms is particularly challenging and important with diverse applications from systems biology to biotechnology. Among the variety of techniques that have been applied, time-of-flight secondary ion mass spectrometry (ToF-SIMS) has many promising features in studying the surface characteristics of biofilms. ToF-SIMS offers high spatial resolution and high mass accuracy, which permit surface sensitive analysis of biofilm components. Thus, ToF-SIMS provides a powerful solution to addressing the challenge of bacterial biofilm analysis. This dataset covers ToF-SIMS analysis of *Paenibacillus sp.* 300A (300A) isolated from the Hanford site in Richland, WA. The strain is known to have metal and sulfur reducing properties and can be used for bioremediation, wastewater treatment, bioengineering and technology development. There is a current need to identify small molecules and fragments produced from bacterial biofilms. Static ToF-SIMS spectra of 300A were obtained using an IONTOF TOF-SIMS V instrument equipped with a 25 keV Bi_3_^+^ metal ion gun. Identified molecules and molecular fragments are compared against known biological databases and the reported peaks have at least 65 ppm mass accuracy. These molecules range from lipids and fatty acids to flavonoids, quinolones, and other naturally occurring organic compounds. It is anticipated that the spectral identification of key peaks will assist detection of metabolites, extracellular polymeric substance molecules like polysaccharides, and biologically relevant small molecules using ToF-SIMS in future surface and interface research of bacterial biofilms.

## Highlights

• Offered ToF-SIMS spectral analysis results of *Paenibacillus sp.* 300A.
• Studied both planktonic cells and biofilms of 300A in ToF-SIMS.
• Identified biological molecules relevant to extracellular polymeric substance.
• Provide spectral reference data of 300A for biofilm research using SIMS.

## Graphical abstract

**Figure:**
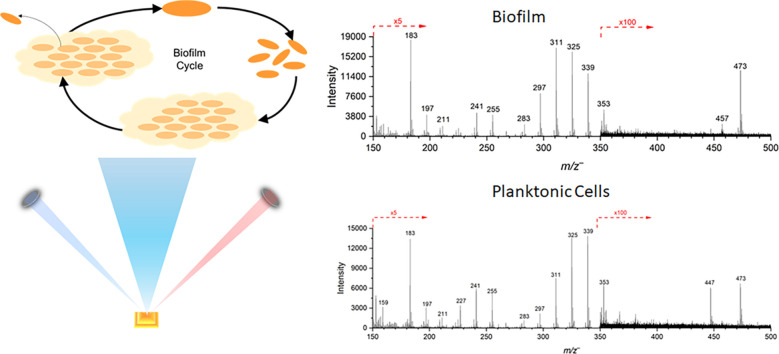

Specifications Table

**Table:** 

Subject	Biological Sciences
Specific subject area	Biochemistry
Type of data	Table; Analyzed Figure; Raw
Data collection	Time-of-flight secondary ion mass spectrometer IONTOF TOF-SIMS V was used to collect data in static mode. Static, time-of-flight secondary ion mass spectrometry (ToF-SIMS) spectra were obtained using an IONTOF TOF.SIMS V equipped with a 25 keV Bi_3_^+^ metal ion gun. The raster size for each region of interest was 500 × 500 µm^2^. The number of scans per spectrum is 60. Spectra requiring use of the electron flood gun were specified for each sample. Peak identifications are based on mass formula with a mass deviation of less than 65 ppm.
Data source location	Institution: Oak Ridge National Laboratory City/Town/Region: Oak Ridge, TN Country: United States of America
Data accessibility	Available via *IEEE DataPort,* DOI: 10.21227/h8vv-hg19 Available via Zenodo: https://zenodo.org/records/15446699
Related research article	*NONE*

## 1 Value of the Data

• Understanding and identifying small molecule production that the bacterial strain possesses are crucial since there is no existing database for biofilms and components analysed by ToF-SIMS.
• *Paenibacillus sp.* 300A comes from the subsurface of the 300 Area of the Hanford site, an area which is contaminated with uranium due to the past nuclear processing at that location [1]. This species is a Gram-positive, rod-shaped facultative anaerobe [1]. Other *Paenibacillus* rhizobacteria species are known for production of biologically relevant compounds, such as nonribosomally formed peptides, polyketides, antibiotics, phytohormones, lytic enzymes, other biocins, and a wide range of exopolysaccharides, which aid in plant growth and antimicrobial effects [2,3]. The authors are unaware of any published reference SIMS mass spectra for this strain.
• Using specific bacteria strains to enhance bioremediation is desirable for industries, like agriculture, nuclear waste management, and healthcare. Identifying compounds that bacterial strains secrete can lead to advancement in diverse applications and support efforts towards building a community database for identification of small molecules inherent of extracellular polymeric substance (EPS) using ToF-SIMS.
• This dataset provides identification of small organic molecules, amino acids, fatty acids, and lipids among other compounds for *Paenibacillus sp.* 300A*.* This data can be used as a control dataset for the specified organism and as a basis for identification of molecules detected in both positive and negative modes.

## 2 Background

*Paenibacillus sp.* 300A comes from the subsurface of the 300 Area of the Hanford site, an area which is contaminated with uranium due past nuclear processing at that location [1]. This species is a Gram-positive, rod-shaped facultative anaerobe [1]. Metals like iron, chromium, and uranium are used as electron acceptors for this bacterium via direct electron transfer or mediated electron transfer [4].

Currently there is no database that identifies small molecules or fragments of products of biofilms via ToF-SIMS for plant growth promotion rhizobacteria (PGPR) species. ToF-SIMS has been used to study biological systems, in particular because of its high mass resolving power and superior surface sensitivity of organics [5,6]. The main objective is to provide a summary of key peaks in both negative and positive ion mode from static ToF-SIMS analysis. ToF-SIMS spectra acquired from both the planktonic cells and corresponding biofilms are both reported. This article focuses mainly on the mid-mass region (*m/z* 150 – 500), which shows detection of metabolites, fatty acids, and fragments of lipids and polysaccharides from planktonic cells and biofilms of the PGPR strain.

## 3 Data Description

**Fig. 1:**
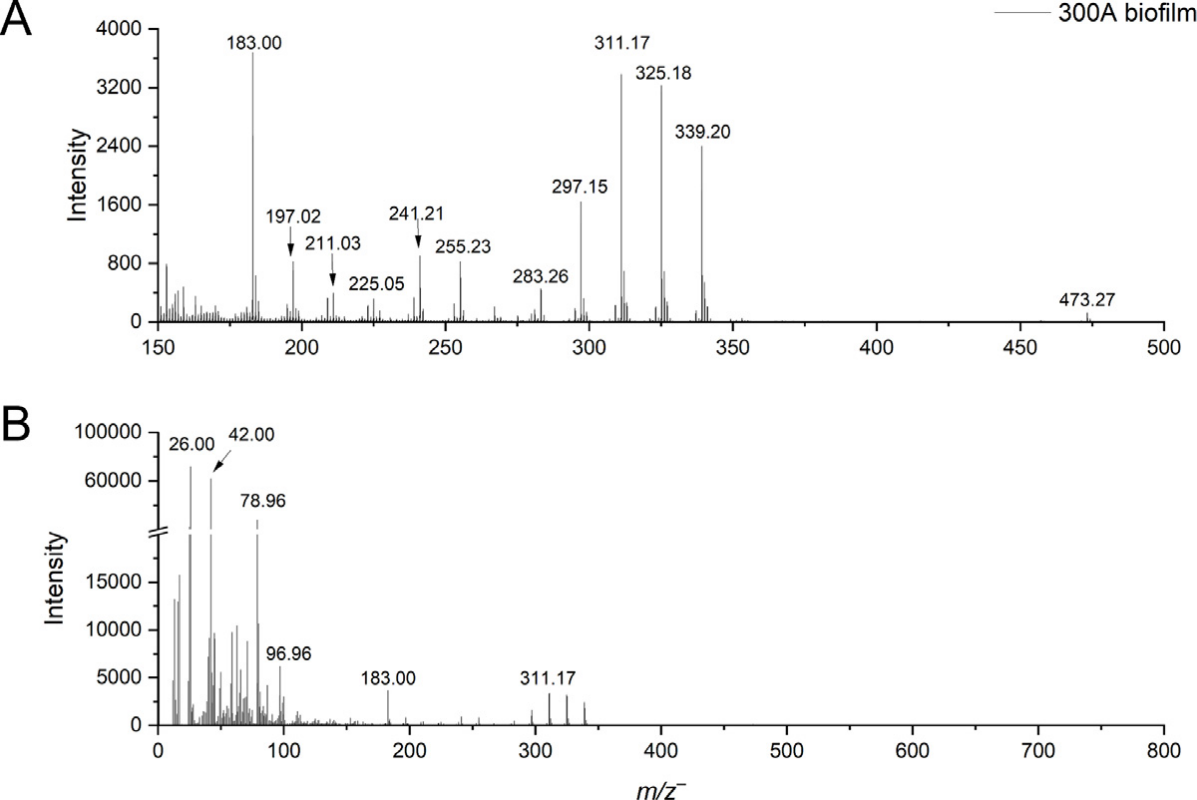
ToF-SIMS spectra of *Paenibacillus sp.* 300A biofilms in the negative ion mode highlighting (a) *m/z*^–^ 150-500 and (b) the total mass spectra *m/z*^–^ 0-800.

**Fig. 2:**
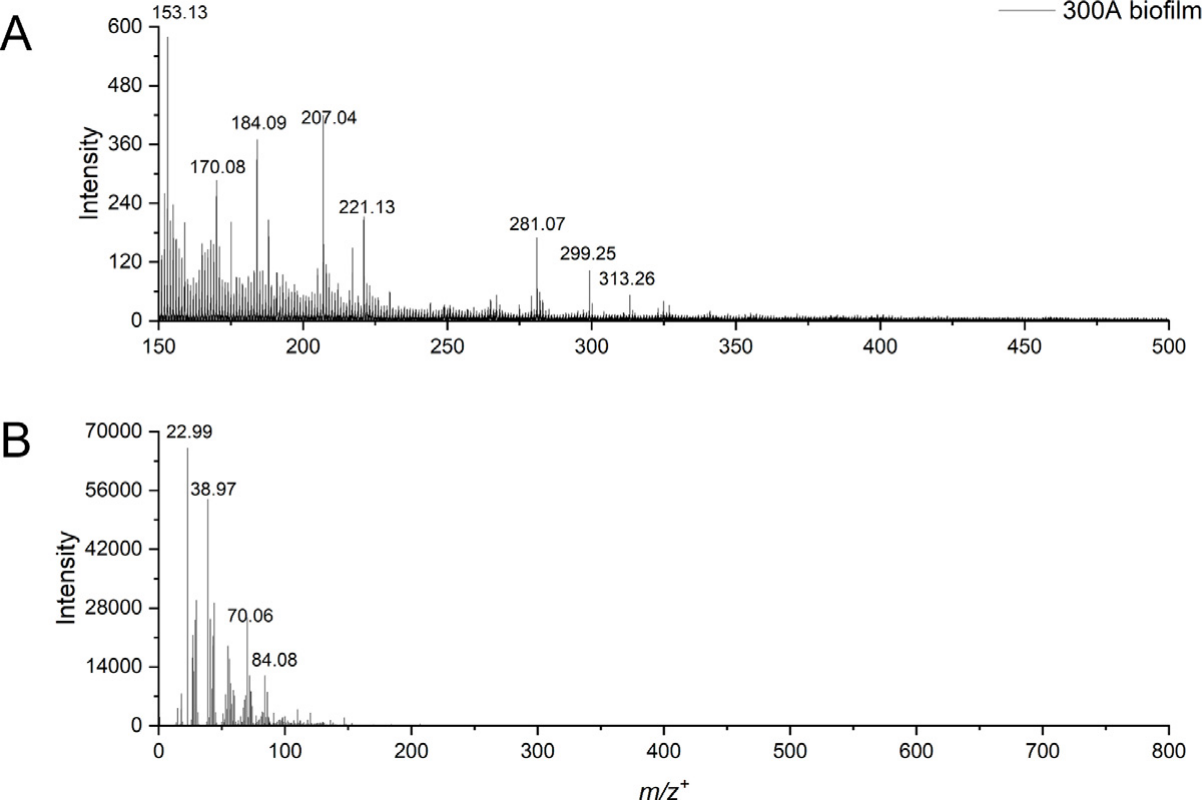
ToF-SIMS spectra of *Paenibacillus sp*. 300A biofilms in the positive ion mode highlighting (a) *m/z*^+^ 150-500 and (b) the total mass spectra *m/z*^+^ 0-800.

**Fig. 3:**
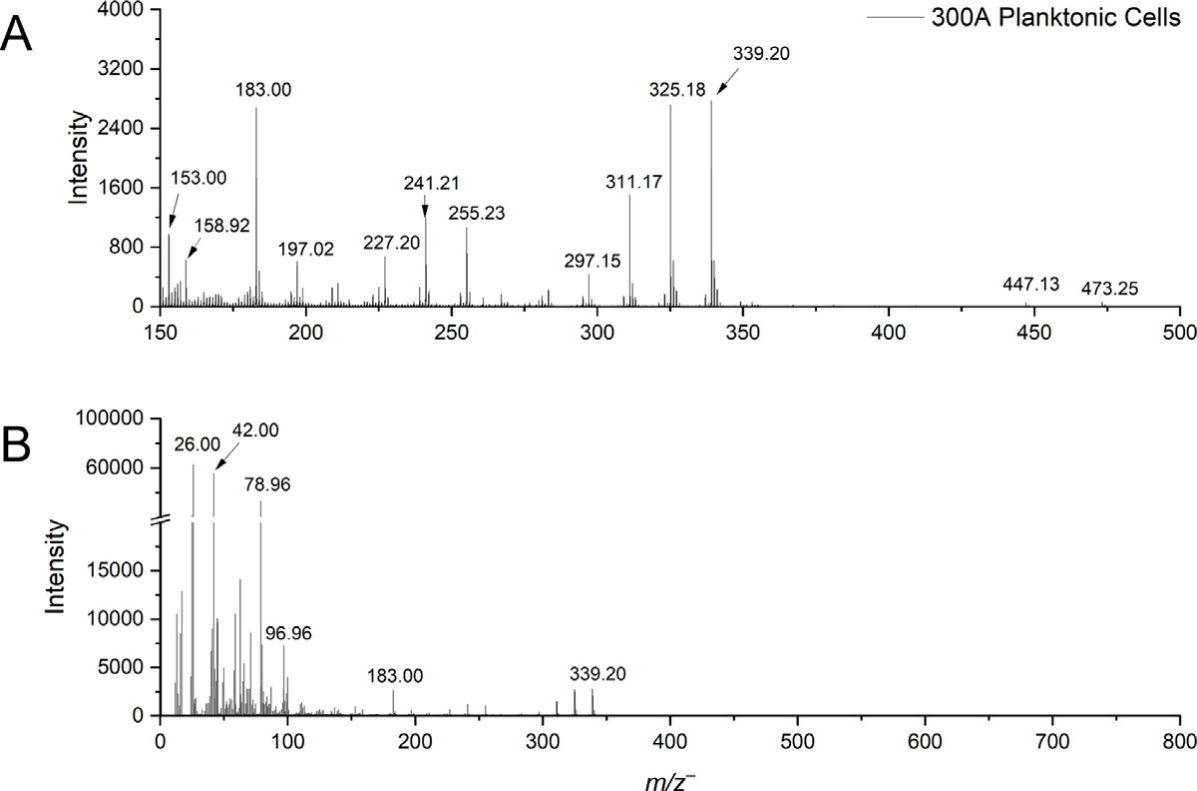
Static ToF-SIMS spectra of *Paenibacillus sp.* 300A planktonic cells in the negative ion mode highlighting (a) *m/z*^–^ 150-500 and (b) the total mass spectra *m/z*^–^ 0-800.

**Fig. 4:**
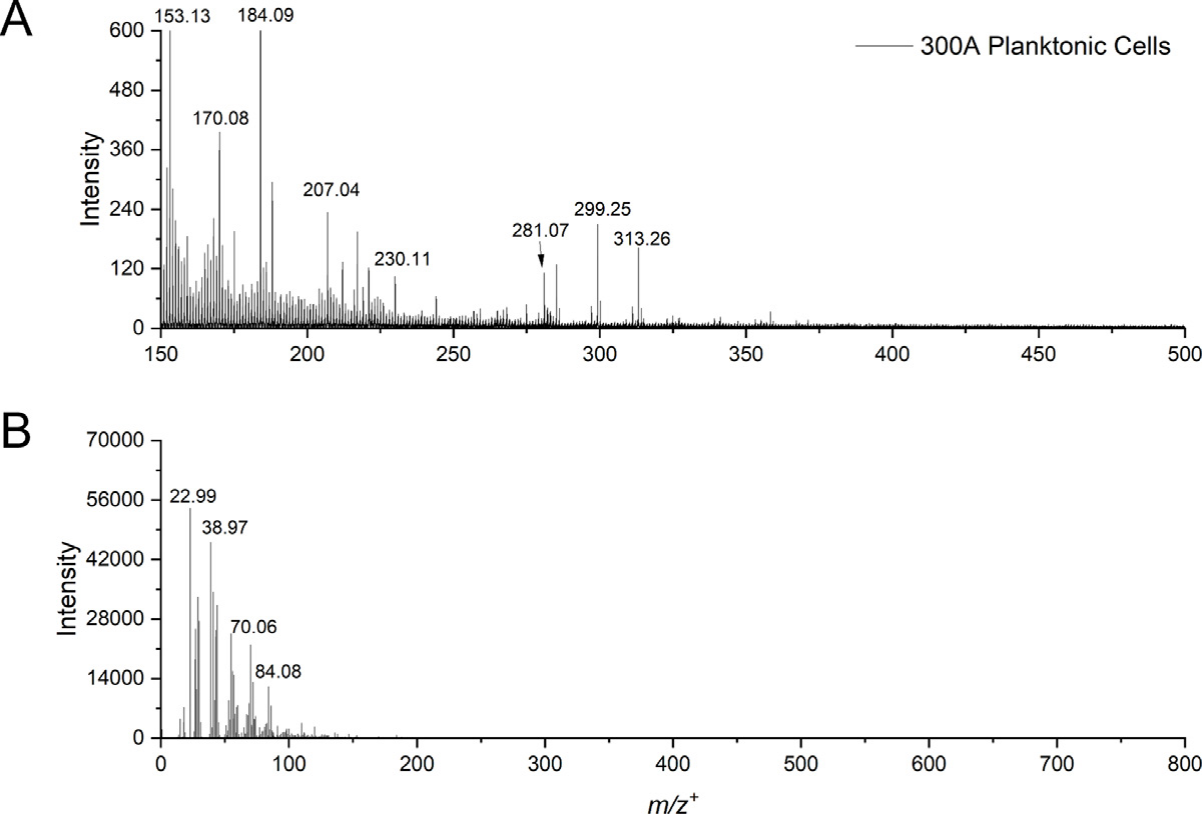
Static ToF-SIMS spectra of *Paenibacillus sp.* 300A planktonic cells in the positive ion mode highlighting (a) *m/z*^+^ 150-500 and (b) the total mass spectra *m/z*^+^ 0-800.

We focus on reporting the molecules with relevance to EPS [7,8]. Collected ToF-SIMS spectra show the mass range of 0-800 Da while highlighting the mass range 150-500 Da, as this region is of interest for metabolite, fatty acid, and lipid production among other molecules. Fig. 1 depicts the ToF-SIMS spectra of 300A biofilms captured in the negative mode. Table 1 shows possible peak identifications for the 300A biofilm in the negative mode. Fig. 2 depicts the spectra of 300A biofilm acquired in the positive mode. Table 2 possible peak identifications for the 300A biofilm in the positive mode. Fig. 3 shows the ToF-SIMS spectra of planktonic cells captured in the negative mode. Table 3 shows possible peak identifications for the 300A planktonic cells in the negative mode. Fig. 4 shows the ToF-SIMS spectra of planktonic cells captured in the positive mode. Table 4 shows possible peak identifications for the 300A planktonic cells in the positive mode. Each mode provides complementary information of the bacteria. We highlight molecules, such as amino acids, phosphates, sulfates, fatty acids, and lipids, which are important EPS components of the 300A bacterial systems. Supplementary Tables S1 – S4 show ToF-SIMS acquisition conditions used for the biofilm and planktonic cell samples and associated key peak identification of the 300A strain, respectively.

**Table 1:** Possible peak identification of molecules of *Paenibacillus sp.* 300A biofilm in the negative ion mode.

*m/z*^−^*_obs._*	*m/z*^−^*_theo._*	∆M, ppm	Species	Assignment	Reference
72.0090	72.0091	-1.6017	$C_{2}H_{2}NO_{2}^{-}$	Amino acid	PubChem CID 3080609
78.9633	78.9591	53.1954	$PO_{3}^{-}$	Phosphite	PubChem CID 107908
87.0087	87.0088	-0.7136	$C_{3}H_{3}O_{3}^{-}$	Alpha-keto acid	RefMet ID: RM0134967
100.0417	100.0404	12.4833	$C_{4}H_{6}NO_{2}^{-}$	Amino acid	RefMetID: RM0132184
121.0301	121.0295	5.1156	$C_{7}H_{5}O_{2}^{-}$	Phenolic acid	RefMet ID: RM0134993
125.0356	125.0357	-0.5697	$C_{5}H_{5}N_{2}O_{2}^{-}$	Nucleic acid	RefMet ID: RM0040235
142.0603	142.0598	3.2031	$C_{3}H_{9}N_{3}O_{2}Na^{-}$	Amino acid, Sodium adduct	PubChem CID 6453265
152.9991	152.9982	5.5450	$C_{4}H_{5}N_{2}O_{2}Ca^{-}$	Amino acid, Calcium adduct	RefMet ID: RM0136807
183.0079	183.0064	7.9735	$C_{4}H_{8}O_{6}P^{-}$	Phosphate ester	RefMet ID: RM0137078
197.0237	197.0207	15.3568	$C_{3}H_{8}N_{3}O_{5}P^{-}$	Amino acid	RefMet ID: RM0136241
211.0399	211.0486	-41.3290	$C_{9}H_{9}NO_{5}^{-}$	Amino acid	RefMet ID: RM0136351
225.0538	225.0643	-46.7388	$C_{10}H_{11}NO_{5}^{-}$	Amino acid	RefMet ID: RM0138700
241.2173	241.2173	0.1676	$C_{15}H_{29}O_{2}^{-}$	Fatty acid	RefMet ID: RM0153573
255.2316	255.2330	-5.2741	$C_{16}H_{31}O_{2}^{-}$	Fatty acid	RefMet ID: RM0153571
267.1031	267.0986	16.7497	$C_{12}H_{15}N_{2}O_{5}^{-}$	Dipeptide	RefMet ID: RM0129807
283.2626	283.2643	-5.6851	$C_{18}H_{35}O_{2}^{-}$	Fatty acid	RefMet ID: RM0153574
297.1567	297.1496	23.8506	$C_{19}H_{21}O_{3}^{-}$	Metabolite	RefMet ID: RM0188879
311.1741	311.1653	28.4618	$C_{20}H_{23}O_{3}^{-}$ or $C_{13}H_{29}O_{6}P^{-}$	Metabolite	RefMet ID: RM0126037 or RM0179668
325.1898	325.1809	27.3603	$C_{21}H_{25}O_{3}^{-}$	Metabolite	RefMet ID: RM0003142
339.2054	339.1966	25.9762	$C_{22}H_{27}O_{3}^{-}$	Metabolite	RefMet ID: RM0071119
353.2198	353.2122	21.4059	$C_{23}H_{29}O_{3}^{-}$	Metabolite	RefMet ID: RM0127981
457.2295	457.2317	-4.8106	$C_{21}H_{29}N_{8}O_{4}^{-}$	Tripeptide	RefMet ID: RM0139177
473.2786	473.2756	6.2903	$C_{24}H_{41}O_{9}^{-}$	Glycerolipid	RefMet ID: RM0084428
501.3124	501.3069	10.9991	$C_{26}H_{45}O_{9}^{-}$	Glycerolipid	RefMet ID: RM0020985

Footnotes:

*m/z*^–^_theo._: theoretical mass to charge ratio in the negative ion mode.

*m/z*^–^_obs._: observed mass to charge ratio in the negative ion mode.

ΔM: ΔM = 10^6^ × (*m/z*^–^_obs._− *m/z*^–^_theo_.)/ *m/z*^–^_theo._ (expressed in ppm) [9,10].

References are provided by PubChem or Metabolomics Workbench.

**Table 2:** Possible peak identification of molecules of the *Paenibacillus sp.* 300A biofilm in the positive ion mode.

*m/z*^+^*_obs._*	*m/z*^+^*_theo._*	∆M, ppm	Species	Assignment	Reference
84.0861	84.0808	63.2974	$C_{5}H_{10}N^{+}$	Hydropyridines	RefMet ID: RM0030080
110.0808	110.0839	-27.9815	$C_{6}H_{10}N_{2}^{+}$	Imidizole	PubChem CID11182587
120.0892	120.0893	-1.3111	$C_{4}H_{12}N_{2}O_{2}^{+}$	Hydroxylamines	PubChem CID99287
147.0830	147.0804	17.7088	$C_{10}H_{11}O^{+}$	Cinnamic acid	RefMet ID: RM0005622
153.1341	153.1274	43.5427	$C_{10}H_{17}O^{+}$	Isoprenoids	RefMet ID: RM0135325
170.0846	170.0937	-54.0296	$C_{9}H_{14}O_{3}^{+}$	Heteroarene	RefMet ID: RM0118125
184.0917	184.0842	40.7099	$C_{8}H_{12}N_{2}O_{3}^{+}$	Cyclic dipeptide	RefMet ID: RM0156123
207.0455	207.0499	-21.5085	$C_{7}H_{11}O_{7}^{+}$	Tricarboxylic acids	RefMet ID: RM0137020
217.1845	217.1798	21.3755	$C_{12}H_{25}O_{3}^{+}$	Fatty acid	RefMet ID: RM0153259
221.1320	221.1271	22.0846	$C_{10}H_{15}N_{5}O^{+}$	Nucleic acids	RefMet ID: RM0136372
230.1137	230.1261	-53.8338	$C_{10}H_{18}N_{2}O_{4}^{+}$	Amino acid	RefMet ID: RM0162202
267.0085	266.9961	46.2977	$C_{7}H_{10}NO_{6}PS^{+}$	Thiazoles	RefMet ID: RM0137098
281.0765	281.0899	-47.6716	$C_{13}H_{15}O_{4}NO_{6}^{-}$	Amino acid	RefMet ID: RM0136301
299.2577	299.2581	-1.4070	$C_{18}H_{35}O_{3}^{+}$	Fatty acid	RefMet ID: RM0134995
313.2670	313.2737	-21.3388	$C_{19}H_{37}O_{3}^{+}$	Fatty acid	RefMet ID: RM0152588
537.4743	537.4877	-24.9653	$C_{34}H_{65}O_{4}^{+}$	Glycerolipids	RefMet ID: RM0075766

Footnotes:

*m/z*^+^_theo._: theoretical mass to charge ratio in the negative ion mode.

*m/z*^+^_obs._: observed mass to charge ratio in the negative ion mode.

ΔM: ΔM = 10^6^ × (*m/z*^+^_obs._− *m/z*^+^_theo_.)/ *m/z*^+^_theo._ (expressed in ppm) [9,10].

**Table 3:** Possible peak identification of molecules of the *Paenibacillus sp*. 300A planktonic cells in the negative ion mode.

*m/z^–^_obs._*	*m/z^–^_theo._*	∆M, ppm	Species	Assignment	References
62.9635	62.9641	-9.8208	$PO_{2}^{-}$	Hypophosphite	PubChem CID 183145
78.9623	78.9591	41.1654	$PO_{3}^{-}$	Phosphite	PubChem CID 107908
96.9730	96.9696	34.6539	$PO_{4}H_{2}^{-}$	Phosphoric acid	PubChem CID 1004
100.0412	100.0404	7.7802	$C_{4}H_{6}NO_{2}^{-}$	Amino acid	RefMetID: RM0132184
113.0410	113.0357	47.6865	$C_{4}H_{5}N_{2}O_{2}^{-}$	Nucleic acid	RefMetID: RM0135879
125.0358	125.0357	1.4286	$C_{5}H_{5}N_{2}O_{2}^{-}$	Nucleic acid	RefMet ID: RM0040235
153.0021	152.9982	25.3987	$C_{4}H_{5}N_{2}O_{2}Ca^{-}$	Nucleic acid, Calcium adduct	RefMet ID: RM0136807
158.9269	158.9254	9.7988	$P_{2}O_{6}H^{-}$	Hypophosphate	PubChem CID 16131857
183.0074	183.0064	5.4330	$C_{4}H_{8}O_{6}P^{-}$	Phosphate ester	RefMet ID: RM0137078
197.0233	197.0207	12.9779	$C_{3}H_{8}N_{3}O_{5}P^{-}$	Amino acid	RefMet ID: RM0136241
211.0402	211.0486	-39.8135	$C_{9}H_{9}NO_{5}^{-}$	Amino acid	RefMet ID: RM0136351
227.2013	227.2017	-1.6694	$C_{14}H_{27}O_{2}^{-}$	Fatty acid	RefMet ID: RM0153483
241.2172	241.2173	-0.6117	$C_{15}H_{29}O_{2}^{-}$	Fatty acid	RefMet ID: RM0153573
255.2320	255.2330	-3.8522	$C_{16}H_{31}O_{2}^{-}$	Fatty acid	RefMet ID: RM0153571
267.1036	267.1027	3.5758	$C_{12}H_{15}N_{2}O_{5}^{-}$	Dipeptide	RefMet ID: RM0129807
283.2610	283.2643	-11.4452	$C_{18}H_{35}O_{2}^{-}$	Fatty acid	RefMet ID: RM0153574
297.1550	297.1496	18.0652	$C_{19}H_{21}O_{3}^{-}$	Metabolite	RefMet ID: RM0188879
311.1717	311.1653	20.7558	$C_{20}H_{23}O_{3}^{-}$ or $C_{13}H_{29}O_{6}P^{-}$	Metabolite	RefMet ID: RM0126037 or RM0179668
325.1889	325.1809	24.6656	$C_{21}H_{25}O_{3}^{-}$	Metabolite	RefMet ID: RM0003142
339.2049	339.1966	24.6956	$C_{22}H_{27}O_{3}^{-}$	Metabolite	RefMet ID: RM0071119
353.2112	353.2122	-2.9081	$C_{23}H_{29}O_{3}^{-}$	Metabolite	RefMet ID: RM0127981
447.1363	447.1297	14.8723	$C_{22}H_{23}O_{10}^{-}$	Flavonoid	RefMet ID: RM0052995
473.2794	473.2756	7.9510	$C_{24}H_{41}O_{9}^{-}$	Glycerolipid	RefMet ID: RM0084428

Footnotes:

*m/z*^–^_theo._: theoretical mass to charge ratio in the negative ion mode.

*m/z*^–^_obs._: observed mass to charge ratio in the negative ion mode.

ΔM: ΔM = 10^6^ × (*m/z*^–^_obs._− *m/z*^–^_theo_.)/ *m/z*^–^_theo._ (expressed in ppm) [9,10].

References are provided by PubChem or Metabolomics Workbench.

**Table 4:** Possible peak identification of molecules of the *Paenibacillus sp*. 300A planktonic cells in the positive ion mode.

*m/z*^+^_obs._	*m/z*^+^_theo._	∆M, ppm	Species	Assignment	References
70.0696	70.0651	63.5526	$C_{4}H_{8}N^{+}$	Pyrrolines	RefMet ID: RM0038534
72.0852	72.0808	61.8313	$C_{4}H_{10}N^{+}$	Pyrrolidine alkaloids	RefMet ID: RM0034922
84.0854	84.0808	54.9601	$C_{5}H_{10}N^{+}$	Hydropyridines	RefMet ID: RM0030080
100.0812	100.0757	55.4188	$C_{5}H_{10}NO^{+}$	Piperidinones	RefMet ID: RM0016041
110.0796	110.0839	-38.5444	$C_{6}H_{10}N_{2}^{+}$	Imidizole	PubChem CID11182587
120.0884	120.0893	-7.8735	$C_{4}H_{12}N_{2}O_{2}^{+}$	Hydroxylamines	PubChem CID 99287
136.0787	136.0730	41.9345	$C_{5}H_{12}O_{4}^{+}$	Monosaccharides	RefMet ID: RM0049554
147.0792	147.0764	19.0584	$C_{5}H_{11}N_{2}O_{3}^{+}$	Dipeptides	RefMet ID: RM0044239
153.1327	153.1274	34.5528	$C_{10}H_{17}O^{+}$	Isoprenoids	RefMet ID: RM0135325
170.0868	170.0937	-40.5900	$C_{9}H_{14}O_{3}^{+}$	Heteroarene	RefMet ID: RM0118125
175.1157	175.1190	-18.7478	$C_{6}H_{15}N_{4}O_{2}^{+}$	Amino acids	RefMet ID: RM0135963
184.0943	184.0968	-13.9091	$C_{9}H_{14}NO_{3}^{+}$	Phenylethylamines	RefMet ID: RM0046981
188.0989	188.1070	-42.7621	$C_{12}H_{14}ON^{+}$	Phenylamino	PubChem CID 5289289
207.0452	207.0499	-22.8705	$C_{7}H_{11}O_{7}^{+}$	Tricarboxylic acids	RefMet ID: RM0137020
217.1807	217.1798	4.0102	$C_{12}H_{25}O_{3}^{+}$	Fatty acid	RefMet ID: RM0153259
230.1131	230.1261	-56.3296	$C_{10}H_{18}N_{2}O_{4}^{+}$	Amino acid	RefMet ID: RM0162202
244.1198	244.1094	42.7400	$C_{15}H_{16}O_{3}^{+}$	Isoprenoids	RefMet ID: RM0137173
259.1604	259.1693	-34.1450	$C_{17}H_{23}O_{2}^{+}$	Fatty alcohols	RefMet ID: RM0150671
268.2560	268.2397	60.9059	$C_{17}H_{32}O_{2}^{+}$	Fatty acids	RefMet ID: RM0152615
281.0713	281.0899	-66.1710	$C_{13}H_{15}O_{4}NO_{6}^{-}$	Amino acid	RefMet ID: RM0136301
285.2355	285.2424	-24.1924	$C_{17}H_{33}O_{3}^{+}$	Fatty acid	RefMet ID: RM0153311
299.2507	299.2581	-24.7521	$C_{18}H_{35}O_{3}^{+}$	Fatty acid	RefMet ID: RM0134995
313.2638	313.2737	-31.6156	$C_{19}H_{37}O_{3}^{+}$	Fatty acid	RefMet ID: RM0152588
358.3448	358.3230	60.8480	$C_{25}H_{42}O^{+}$	Sterol Lipids	RefMet ID: RM0128098
509.4379	509.4564	-36.3069	$C_{32}H_{61}O_{4}^{+}$	Glycerolipids	RefMet ID: RM0076179
523.4458	523.4721	-50.3065	$C_{33}H_{63}O_{4}^{+}$	Glycerolipids	RefMet ID: RM0076191
537.4592	537.4877	-53.0280	$C_{34}H_{65}O_{4}^{+}$	Glycerolipids	RefMet ID: RM0075766
563.4324	563.4670	-61.4791	$C_{35}H_{63}O_{5}^{+}$	Glycerolipids	RefMet ID: RM0035447
577.4530	577.4827	-51.3504	$C_{36}H_{65}O_{5}^{+}$	Glycerolipids	RefMet ID: RM0034829

Footnotes:

*m/z*^+^_theo._: theoretical mass to charge ratio in the negative ion mode.

*m/z*^+^_obs._: observed mass to charge ratio in the negative ion mode.

ΔM: ΔM = 10^6^ × (*m/z*^+^_obs._− *m/z*^+^_theo_.)/ *m/z*^+^_theo._ (expressed in ppm) [9,10].

## 4 Experimental Design, Materials and Methods

The *Paenibacillus* bacteria strain was cultured on Tryptic Soy Broth (TSB) agar plates [11,12]. All agar plates were incubated at 30°C for 24 hours. After assessing cultures for contamination, one or two pure colonies were inoculated in 10 mL of TSB medium. Planktonic cells were grown to ∼0.6 optical density (OD_600_) [4,13]. Planktonic cells were then harvested by centrifugation for 5 min. at 5000 rpm. After centrifugation, the supernatant was discarded and replaced with 1 mL sterile deionized (DI) water to resuspend. This process was repeated three times, then 200 µL DI water was added for a final resuspension. The planktonic cells were then plated onto sterilized silicon (Si) wafers and air dried under laminar flow within a biosafety cabinet (BSC) [14]. Biofilms were cultured using static cells described previously [15]. Biofilms were grown for 5-6 days, and maturation was observed via optical microscope. Biofilms were then plated onto sterilized Si wafers and dried under laminar flow within a BSC. Si wafer controls were prepared by sonication of wafers in 30 mL ethanol, isopropanol, and acetone, respectively for 5 minutes. The wafers were then blown dry with nitrogen gas after each sonication bath. The cleaned Si wafers were then treated with UV-ozone (model No. 342, Jetlight Company Inc.) for one minute to render the surface hydrophilic [4,15].

Static ToF-SIMS spectra were obtained using an IONTOF TOF-SIMS V equipped with a 25 keV Bi_3_^+^ metal ion gun. The static limit is defined as < 10^13^ ions/cm^2^. Here we calculate the primary ion dose density to be 6.90 × 10^11^ ions/cm^2^. The spectral pixel resolution was 128 × 128. The pulse length was 25 ns and the static primary current was 27 nA. The spectral data was collected with 1 pulse/pixel. The raster size for each region of interest was 500 × 500 µm. Normally, a large area is used when the sample is relatively flat and uniform. The number of scans per spectra is 25. Spectra requiring use of the electron flood gun is specified for each sample. The measurement cycle time was 100 µs ensuring data collections past *m/z* 800. The figures shown here have possible identifications of molecules matching software (IONTOF *SurfaceSpectra v7*) suggested mass formula with a mass deviation of less than 65 ppm. The SIMS mass accuracy is defined as ∆M: = 10^6^ × (*m/z*_obs_ − *m/z*_the_)/ *m/z*_the_ (expressed in ppm), where *m/z*_obs_ and *m/z*_the_ refer to the observed and theoretical mass to charge ratio of a specific peak in the negative or positive ion mode [9,10].

The identification procedure followed a multi-step process. The IONTOF database with IonTOF peak searching and mass matching functions are used in peak identification. External database searches were also performed to ascertain peak determination using PubChem, KEGG, and MetabolomicsWorkbench. The mass matching function calculates different combinations of periodic table elements to mass match a selected peak and it will provide a mass deviation and match score. These combinations range from organics to inorganics. Using the “peak search” function within *SurfaceSpectra*, with parameters of SNR 3.0, max background 0.8, and minimum counts 25, a peak list was generated giving the best mass matching formula for the given *m/z* value under each specific sample. The peak center mass is entered into external databases to identify biologically relevant possible matches. The bacterial system, isotopic ratio, and plausibility for each identified peak are considered for assignment.

Media controls were also analysed and given peak lists for comparison. Sample and media control peak lists were exported into Excel® where the sample values and media values were sorted based on mass values. Sample peak values within ± *m/z* 0.05 of media peak values were not included in the provided tables unless the peak has substantial intensity. This value of ± *m/z* 0.05 was chosen to compensate for slight variations between analysis of sample and media. The biofilm or planktonic sample had some overlapping spectral features to with media. If there were standout values above ± *m/z* 0.05, then they were treated as unique to the biofilm or planktonic cell sample. These removals left biological peaks not relating to media or other inorganic artifacts.

Peak assignment was further verified by literature search pertaining to possible biological molecules. Many databases were surveyed for molecular assessment such as PubChem, ChEBI, LipidMaps, MetabolomicsWorkbench, and KEGG. Assignment was based on the mass accuracy from the associated peak value to the corresponding value within the aforementioned databases. All identifications are molecular classes, such as fatty acid, lipid, metabolite, etc., only due to the large number of possibilities each *m/z* value presents, with the best match based on relative mass accuracy and possible biofilm biological functions. While mass matching is important in peak identification, it is not the only factor considered when assigning peaks. For example, *m/z^–^* 311.1741 is identified as C_20_H_23_O_3_^–^, however, it also has the possibility to be C_12_H_21_N_7_O_3_^—^, C_15_H_25_N_3_O_4_^—^, or C_13_H_21_N_5_O_4_^—^. For meaningful peak assignment, we take into consideration factors such as the isotopic ratios, if present, the surrounding spectra and biological context, and the ionization possibility of the molecule. For example, while the mass values for C_12_H_21_N_7_O_3_^—^, C_15_H_25_N_3_O_4_^—^, and C_13_H_21_N_5_O_4_^—^ match closely with 311.1741, these molecules are peptides in their neutral state. On the other hand, C_20_H_23_O_3_^—^ is the anion of C_20_H_24_O_3_, which is a hydroxybenzoate enzyme molecule. When considering the charge of the molecule, there are three other possibilities which the mass 311.1741 could be labelled. It could be C_13_H_28_O_6_P^—^, C_17_H_27_O_3_S^—^, or C_19_H_23_N_2_O_2_^—^. C_13_H_28_O_6_P^—^ is a glycerophospholipid and could be a possibility given the nature of biofilms, C_17_H_27_O_3_S^—^ is a benzenoid benzenesulfonic acid which has a surfactant role, and C_19_H_23_N_2_O_2_^—^ is a cyclic dipeptide consisting of two arginine. When all things are considered, C_20_H_23_O_3_^—^ and C_13_H_28_O_6_P^—^ are the likely candidates based on available information and our understanding of the biofilms. The chemistry of the molecule is then considered. Due to limited reporting on C_13_H_28_O_6_P^—^, this molecule is not reliable to use in peak assignment. This is why we choose to report C_20_H_23_O_3_^—^ as the possible identification. Major species identified in the 300 A spectra are amino acids, fatty acids, lipids, and other relevant molecules. Our findings are in agreement with previous results [4,16]. More specific identification of molecules would need to be corroborated with tandem MS and/or other complimentary techniques [17]. The same process was applied to *m/z* values below 150, where more inorganic ions are present.

Data plotting was done using OriginPro 2023, where the data was extracted from the SIMS files and converted to ASCII files with bin equal to 1 to show raw data. Data files will be made accessible to the public using data archives. The data was calibrated before ASCII conversion. Calibration points for each of the biological samples was attempted to be made the same when possible. Calibration peaks used for the 300A strain were CH_2_^–^, CHO_2_^–^, C_2_H_2_NO_2_^–^ C_4_H_6_NO_2_^–^, C_15_H_29_O_2_^–^, and C_16_H_31_O_2_^–^ for the negative mode and CH_3_^+^, C_4_H_5_^+^, C_3_H_6_NO_2_^+^, C_8_H_10_NO^+^, C_9_H_14_NO_3_^+^, and C_9_H_16_N_3_O_4_^+^ for positive mode, respectively.

## Limitations

None.

## Ethics Statement

The authors confirm that they have read and follow the ethical requirements for publication in Data in Brief and confirm that the current work does not involve human subjects, animal experiments, or any data collected from social media platforms.

## Credit Author Statement

**Gabriel D. Parker:** Data Curation, Formal Analysis, Investigation, Methodology, Validation, Original Draft Preparation, Writing – review & editing. **Xiao-Ying Yu:** Conceptualization, Funding Acquisition, Data Curation, Formal Analysis, Investigation, Methodology, Project Administration, Resources, Software, Supervision, Validation, Writing – review & editing. **Luke Hanley:** Resources, Supervision, Validation, Writing – review & editing. **Andrew Plymale:** Resources, Supervision, Validation, Writing – review & editing.

## Data Availability

IEEE DataPortToF-SIMS data analysis of Paenibacillus sp. 300A and Shewanella oneidensis MR-1 biofilms (Original data)

## Appendix Supplementary materials

Image, application 1
