# Supplementary material for: ToF-SIMS spectral data analysis of Paenibacillus sp. 300A biofilms and planktonic cells
Source: Data Brief. 2025 Jun 9;61:111763. doi: 10.1016/j.dib.2025.111763 (PMC12221530; doi:10.1016/j.dib.2025.111763)
Supplement: Supplementary file 1 [file mmc1.docx]

Article Title

ToF-SIMS spectral data analysis of *Paenibacillus sp.* 300A biofilms and planktonic cells

**Authors**

Gabriel D. Parker^1,2^, Andrew Plymale^3^, Luke Hanley^1^, and Xiao-Ying Yu^2,^ *

**Affiliations**

1. Department of Chemistry, University of Illinois Chicago, Chicago, IL 60607.

2. Materials Science and Technology Division, Oak Ridge National Laboratory, Oak Ridge, TN 37380.

3. Energy and Environment Directorate, Pacific Northwest National Laboratory, Richland, WA 99352.

**Corresponding author(s)**

Dr. Xiao-Ying Yu (yuxiaoying@ornl.gov)

Table of Contents

[**Additional Figures** S-3](#_Toc198559912)

[**Figure S1:** Biofilm surface topography as shown via standard phone camera capture (a), optical microscope (b), and secondary electron detection via ToF-SIMS (c-d). S-3](#_Toc198559913)

[**Figure S2**: ToF-SIMS spectra for the tryptic soy broth (TSB) media used during this experiment presented in negative mode (a) and positive mode (b). S-4](#_Toc198559914)

[**Figure S3:** Normalized ToF-SIMS spectra displaying the biofilm data compared to the media data in negative mode. S-5](#_Toc198559915)

[**Additional Tables** S-6](#_Toc198559916)

[**Table S1:** ToF-SIMS spectral acquisition parameters used for *Paenibacillus sp. 300A* biofilm in the negative ion mode. S-6](#_Toc198559917)

[**Table S2:** ToF-SIMS spectral acquisition parameters used for *Paenibacillus sp. 300A* biofilm in the positive ion mode. S-7](#_Toc198559918)

[**Table S3:** ToF-SIMS spectral acquisition parameters used for *Paenibacillus sp. 300A* planktonic cells in the negative ion mode. S-8](#_Toc198559919)

[**Table S4:** ToF-SIMS spectral acquisition parameters used for *Paenibacillus sp. 300A* planktonic cells in the positive ion mode. S-9](#_Toc198559920)

[**Table S5:** ToF-SIMS spectral peaks observed for TSB media in the negative mode. S-10](#_Toc198559921)

[**Table S6:** ToF-SIMS spectral peaks observed for TSB media in the positive mode. S-12](#_Toc198559922)

**Additional Figures**

**
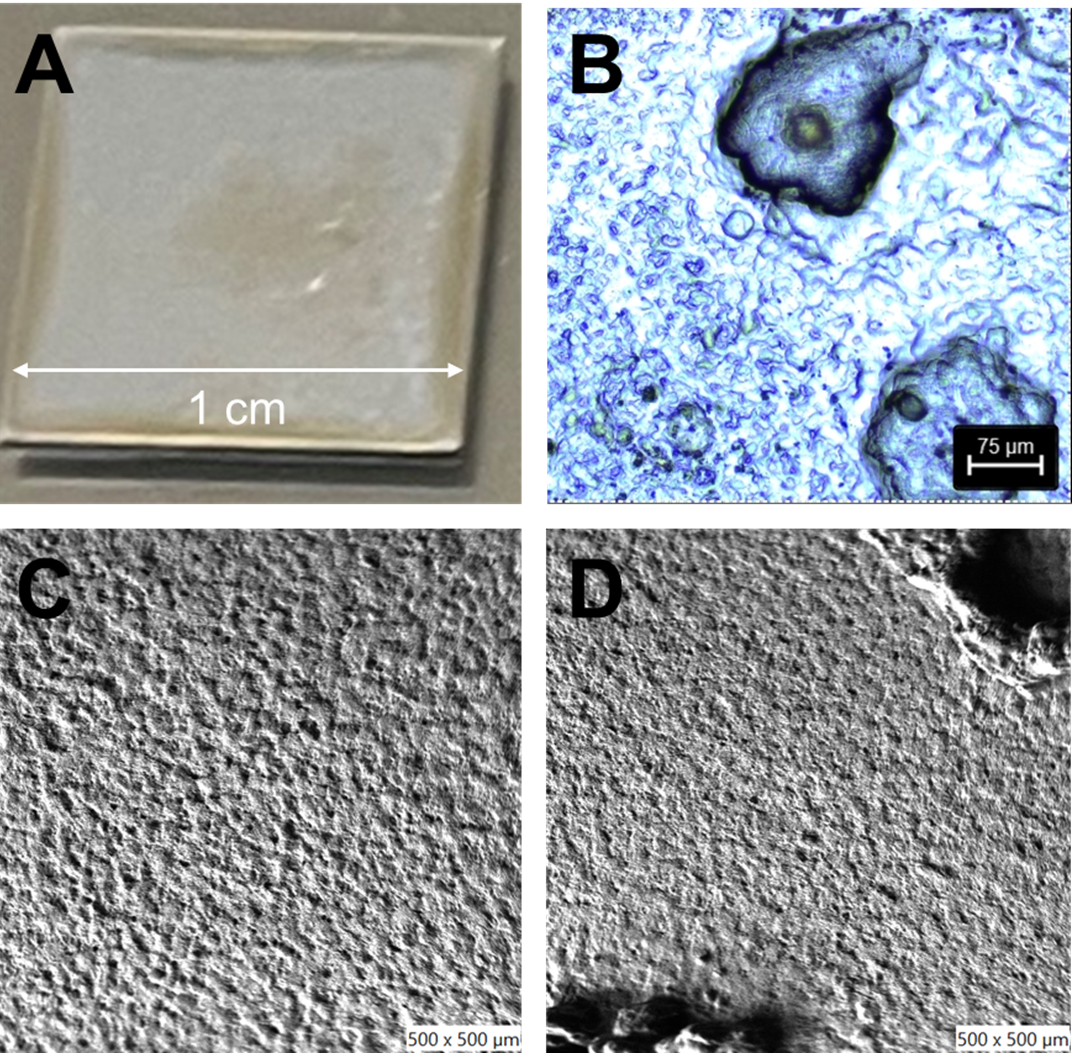
**

**Figure S1:** Biofilm surface topography as shown via standard phone camera capture (a), optical microscope (b), and secondary electron detection via ToF-SIMS (c-d).

**Figure S1** shows the surface topography of representative biofilms analyzed within the main text. **Figure S1a** shows the biofilm surface after desalination and drying on a Si wafer chip captured via a standard phone camera. Biofilms were recast onto the substrate carefully to make a uniform surface as much as possible. Optical imaging via microscope, **Figure S1b**, shows that the surface is relatively uniform with biomass formation. Taking this into account, we observe the biofilm surface as shown in **Figure S1c** and **Figure S1d** via ToF-SIMS secondary electron imaging, similar to those in a scanning electron microscope. Scanning across sample surface was conducted prior to obtaining data. Typical spots that were analyzed are depicted in **Figure S1c**, showing a flat feature. Surface such as **Figure S1d**, while mostly flat, were avoided in SIMS analysis, because it has visible areas that were not covered with biofilms. The uniformity and the topography of the biofilm surface should have no effect on the acquired data as indicated in the secondary electron and optical images.


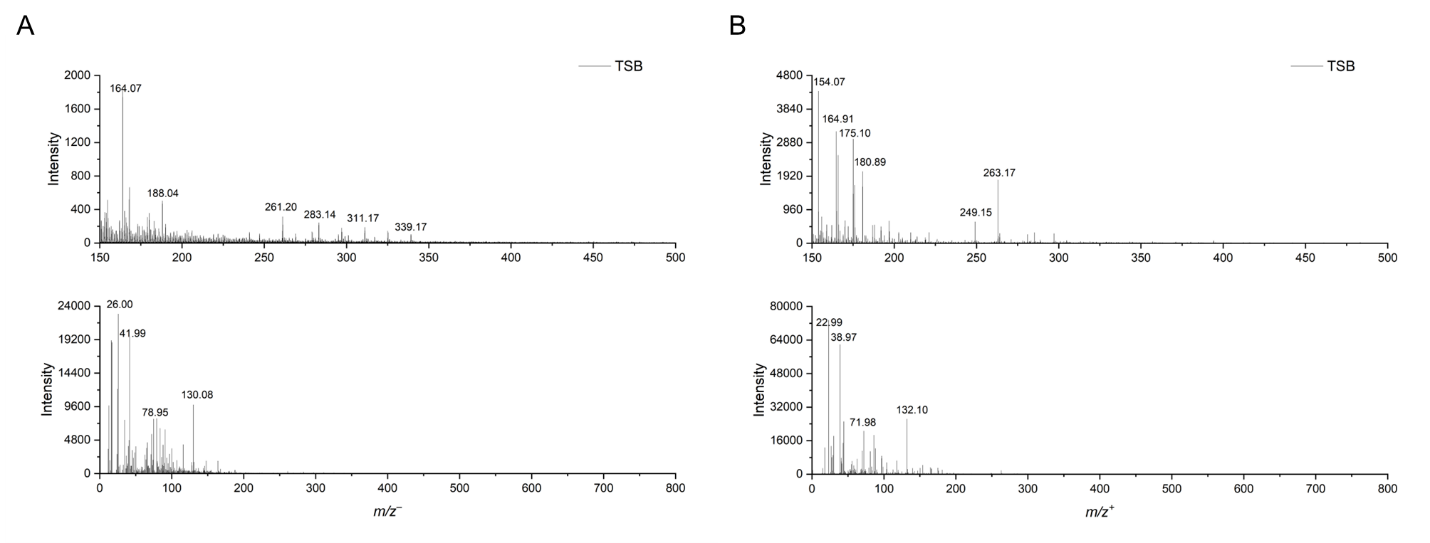


**Figure S2**: ToF-SIMS spectra for the tryptic soy broth (TSB) media used during this experiment presented in negative mode (a) and positive mode (b).

**Figure S2** shows the mass spectra for the tryptic soy broth (TSB) media which was used to culture the biofilms for this experiment. Identification tables for both negative and positive mode data are shown below.


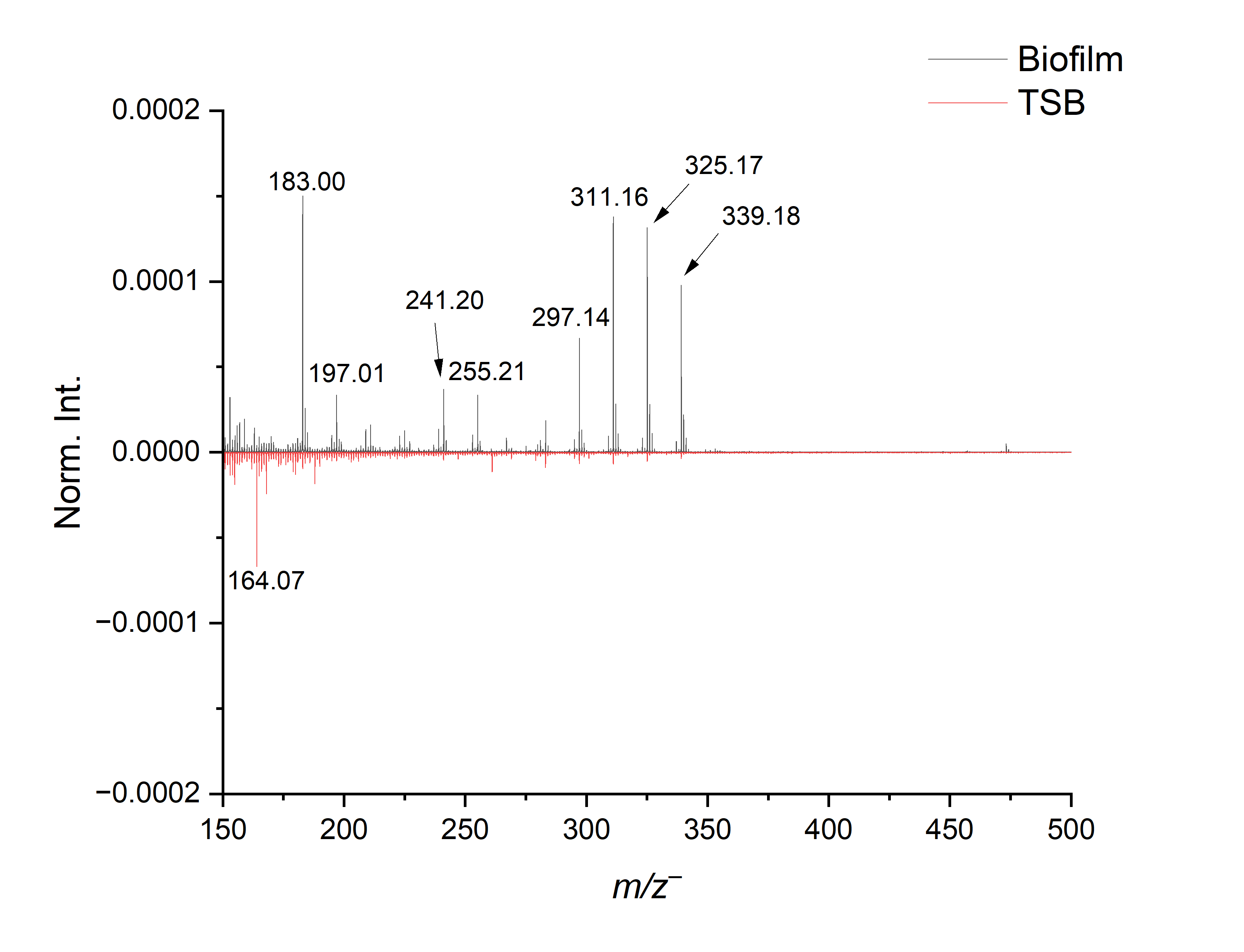


**Figure S3:** Normalized ToF-SIMS spectra displaying the biofilm data compared to the media data in negative mode.

**Figure S3** shows normalized biofilm data and media data for direct comparison of peaks. While some of the media peaks have the same *m/z* values as observed within the biofilm, the biofilm peaks such as *m/z 311.16, 325.17, and 339.18* have increased ion intensity indicating biological material and no media presence. Tryptic soy broth (TSB) does contain biologically relevant molecules within its composition. These peaks observed above the *m/z 300* region are possibly lipids. Increased *m/z* intensity during normalization indicates that the observed peaks are different from the media.

**Additional Tables**

The supplementary tables provided here cover the ToF-SIMS instrument parameters used to collect the static SIMS spectra. They support the peak identification tables and the spectral results of the 300A planktonic cells and biofilms described in the main text.

## **Table S1:** ToF-SIMS spectral acquisition parameters used for *Paenibacillus sp. 300A* biofilm in the negative ion mode.

| Sample | *Paenibacillus sp. 300A* biofilm |
| --- | --- |
| Instrument | IONTOF ToF-SIMS V |
| Mass Analyzer | Time-of-Flight |
| Analyzer Mass Resolution | 4583 m/∆m |
| Mass Resolution m/z value | 59.0148; C_2_H_3_O_2_^–^ |
| Calibration Species | CH_2_^–^, CHO_2_^–^, C_2_H_2_NO_2_^–^ C_4_H_6_NO_2_^–^, C_15_H_29_O_2_^–^, C_16_H_31_O_2_^–^ |
| Primary Ion Gun | Liquid Metal Ion Gun |
| Primary Ion Source | Bi_3_^+^ |
| Primary Ion Dose | 6.902 x 10^11^ ion/cm^2^ |
| Pulse Width | 25.0 ns |
| Pulse Rate | 10.0 kHz |
| Beam Diameter | 5 µm |
| Analysis Area | 500 µm × 500 µm |
| Polarity | Negative |
| Mass Range | 0-800 |
| Spectra Acquisition Time | 40.96 s |

## **Table S2:** ToF-SIMS spectral acquisition parameters used for *Paenibacillus sp. 300A* biofilm in the positive ion mode.

| Sample | *Paenibacillus sp. 300A* biofilm |
| --- | --- |
| Instrument | IONTOF ToF-SIMS V |
| Mass Analyzer | Time-of-Flight |
| Analyzer Mass Resolution | 4507 m/∆m |
| Mass Resolution m/z value | 27.0233; C_2_H_3_^+^ |
| Calibration Species | CH_3_^+^, C_4_H_5_^+^, C_3_H_6_NO_2_^+^, C_8_H_10_NO^+^, C_9_H_14_NO_3_^+^, C_9_H_16_N_3_O_4_^+^ |
| Primary Ion Gun | Liquid Metal Ion Gun |
| Primary Ion Source | Bi_3_^+^ |
| Primary Ion Dose | 6.902 x 10^11^ ion/cm^2^ |
| Pulse Width | 25.0 ns |
| Pulse Rate | 10.0 kHz |
| Beam Diameter | 5 µm |
| Analysis Area | 500 µm × 500 µm |
| Polarity | Positive |
| Mass Range | 0-800 |
| Spectra Acquisition Time | 40.96 s |

## **Table S3:** ToF-SIMS spectral acquisition parameters used for *Paenibacillus sp. 300A* planktonic cells in the negative ion mode.

| Sample | *Paenibacillus sp. 300A* planktonic cells |
| --- | --- |
| Instrument | IONTOF ToF-SIMS V |
| Mass Analyzer | Time-of-Flight |
| Analyzer Mass Resolution | 4812 m/∆m |
| Mass Resolution m/z value | 59.0142; C_2_H_3_O_2_^–^ |
| Calibration Species | CH_2_^–^, CHO_2_^–^, C_2_H_2_NO_2_^–^, C_4_H_6_NO_2_^–^, C_15_H_29_O_2_^–^, C_16_H_31_O_2_^–^ |
| Primary Ion Gun | Liquid Metal Ion Gun |
| Primary Ion Source | Bi_3_^+^ |
| Primary Ion Dose | 6.902 x 10^11^ ion/cm^2^ |
| Pulse Width | 25.0 ns |
| Pulse Rate | 10.0 kHz |
| Beam Diameter | 5 µm |
| Analysis Area | 500 µm × 500 µm |
| Polarity | Negative |
| Mass Range | 0-800 |
| Spectra Acquisition Time | 40.96 s |

## **Table S4:** ToF-SIMS spectral acquisition parameters used for *Paenibacillus sp. 300A* planktonic cells in the positive ion mode.

| Sample | *Paenibacillus sp. 300A* planktonic cells |
| --- | --- |
| Instrument | IONTOF ToF-SIMS V |
| Mass Analyzer | Time-of-Flight |
| Analyzer Mass Resolution | 4477 m/∆m |
| Mass Resolution m/z value | 27.0234; C_2_H_3_^+^ |
| Calibration Species | CH_3_^+^, C_4_H_5_^+^, C_3_H_6_NO_2_^+^, C_8_H_10_NO^+^, C_9_H_14_NO_3_^+^, C_9_H_16_N_3_O_4_^+^ |
| Primary Ion Gun | Liquid Metal Ion Gun |
| Primary Ion Source | Bi_3_^+^ |
| Primary Ion Dose | 6.902 x 10^11^ ion/cm^2^ |
| Pulse Width | 25.0 ns |
| Pulse Rate | 10.0 kHz |
| Beam Diameter | 5 µm |
| Analysis Area | 500 µm × 500 µm |
| Polarity | Positive |
| Mass Range | 0-800 |
| Spectra Acquisition Time | 40.96 s |

## **Table S5:** ToF-SIMS spectral peaks observed for TSB media in the negative mode.

| *m/z*^−^*_obs._* | ∆M, ppm | Species | *m/z*^−^*_obs._* | ∆M, ppm | Species |
| --- | --- | --- | --- | --- | --- |
| $15.023554$ | $-31.238525$ | $CH_{3}^{-}$ | $117.061372$ | $-3.389328$ | $C_{5}H_{11}SN^{-}$ |
| $23.996772$ | $-57.153149$ | $NaH^{-}$ | $118.048079$ | $-24.46277$ | $C_{4}H_{8}NO_{3}^{-}$ |
| $28.017724$ | $-55.269622$ | $CH_{2}N^{-}$ | $119.040728$ | $-2.56683$ | $C_{4}H_{9}SNO^{-}$ |
| $29.003137$ | $-5.229896$ | $CHO^{-}$ | $122.002721$ | $-2.04821$ | $CH_{4}SN_{3}O_{2}^{-}$ |
| $29.994342$ | $40.581434$ | $SiH_{2}^{-}$ | $122.990554$ | $-3.70336$ | $C_{6}H_{3}SO^{-}$ |
| $30.035709$ | $26.174229$ | $CH_{4}N^{-}$ | $129.068561$ | $12.47518$ | $C_{5}H_{9}N_{2}O_{2}^{-}$ |
| $31.971254$ | $-42.702503$ | $S^{-}$ | $131.083548$ | $7.220237$ | $C_{5}H_{11}N_{2}O_{2}^{-}$ |
| $32.980312$ | $-4.025508$ | $HS^{-}$ | $132.981802$ | $-5.77512$ | $CHSN_{4}O_{2}^{-}$ |
| $33.969079$ | $19.534494$ | ^34^$S^{-}$ | $135.030553$ | $-5.04431$ | $C_{5}H_{3}N_{4}O^{-}$ |
| $34.970293$ | $25.494561$ | $Cl^{-}$ | $136.015346$ | $0.703428$ | $C_{5}H_{2}N_{3}O_{2}^{-}$ |
| $35.997042$ | $-30.593266$ | $CHNa^{-}$ | $136.92154$ | $-8.42546$ | $CaSO_{4}H^{-}$ |
| $36.965976$ | $-12.856401$ | ^37^$Cl^{-}$ | $139.038447$ | $-1.99885$ | $C_{5}H_{5}N_{3}O_{2}^{-}$ |
| $37.004821$ | $-30.991153$ | $CH_{2}Na^{-}$ | $140.00975$ | $-2.9636$ | $C_{4}H_{2}N_{3}O_{3}^{-}$ |
| $39.017196$ | $24.890424$ | *CHO^10^B^−^* | $141.045093$ | $-0.54483$ | $CH_{9}SN_{4}O_{2}^{-}$ |
| $43.98734$ | $-54.787925$ | $BHS^{-}$ | $143.072969$ | $-7.53764$ | $C_{10}H_{9}N^{-}$ |
| $44.016595$ | $54.712153$ | $CH_{2}NO^{-}$ | $144.974981$ | $-2.60978$ | $C_{8}HSO^{-}$ |
| $44.997446$ | $-2.868046$ | $BH_{2}S^{-}$ | $145.096083$ | $-5.69031$ | $C_{4}H_{11}N_{5}O^{-}$ |
| $45.989583$ | $28.563399$ | $CH_{2}S^{-}$ | $145.95973$ | $-2.26345$ | $CS_{2}N_{5}^{-}$ |
| $46.996818$ | $15.397872$ | $CH_{3}S^{-}$ | $146.047946$ | $-4.21566$ | $C_{8}H_{6}N_{2}O^{-}$ |
| $49.001014$ | $37.67061$ | $SNH_{3}^{-}$ | $147.008099$ | $-4.54571$ | $C_{8}H_{3}O_{3}^{-}$ |
| $50.003117$ | $-10.103998$ | $C_{3}N^{-}$ | $150.032379$ | $0.905765$ | $C_{8}H_{6}O_{3}^{-}$ |
| $51.017287$ | $-44.426909$ | $C_{3}^{13}CH_{2}^{-}$ | $151.037891$ | $-14.4079$ | $C_{8}H_{7}O_{3}^{-}$ |
| $53.011536$ | $-11.391266$ | $CH_{3}NMg^{-}$ | $152.027732$ | $6.369726$ | $C_{11}H_{4}O^{-}$ |
| $54.034607$ | $-5.84354$ | $C_{3}H_{4}N^{-}$ | $152.895915$ | $5.691501$ | $FeSO_{4}H^{-}$ |
| $55.018732$ | $-3.751459$ | $C_{3}H_{3}O^{-}$ | $153.055925$ | $1.354518$ | $C_{8}H_{9}O_{3}^{-}$ |
| $55.969753$ | $-8.231182$ | $NaHS^{-}$ | $155.08465$ | $-12.7285$ | $C_{12}H_{11}^{-}$ |
| $56.014252$ | $1.162527$ | $C_{2}H_{2}NO^{-}$ | $156.02277$ | $6.998729$ | $C_{10}H_{4}O_{2}^{-}$ |
| $56.05377$ | $57.043656$ | $C_{3}H_{6}N^{-}$ | $157.917665$ | $0.667349$ | $P_{2}O_{6}^{-}$ |
| $56.981354$ | $15.957483$ | $C_{2}HS^{-}$ | $159.00907$ | $1.899364$ | $C_{9}H_{3}O_{3}^{-}$ |
| $57.03262$ | $-34.508601$ | $C_{3}H_{5}O^{-}$ | $160.035438$ | $-31.0158$ | $C_{9}H_{6}NO_{2}^{-}$ |
| $58.032214$ | $40.954239$ | $C_{2}H_{4}NO^{-}$ | $161.99472$ | $-7.02017$ | $C_{8}H_{2}O_{4}^{-}$ |
| $59.013241$ | $-10.374139$ | $C_{2}H_{3}O_{2}^{-}$ | $163.030747$ | $36.40693$ | $C_{5}H_{7}O_{6}^{-}$ |
| $59.952516$ | $-61.79863$ | $Na^{37}Cl^{-}$ | $164.076557$ | $29.5904$ | $C_{9}H_{10}NO_{2}^{-}$ |
| $62.964857$ | $8.593707$ | $COCl^{-}$ | $164.933645$ | $15.41671$ | $Na_{3}PO_{4}H^{-}$ |
| $63.01284$ | $3.959633$ | $CH_{2}NOF^{-}$ | $165.070111$ | $-5.22922$ | $C_{13}H_{9}^{-}$ |
| $63.961865$ | $-9.117157$ | $SO_{2}^{-}$ | $166.055026$ | $8.304476$ | $C_{11}H_{6}N_{2}^{-}$ |
| $64.014378$ | $21.455461$ | $C_{2}H_{2}F_{2}^{-}$ | $167.044432$ | $-22.8178$ | $C_{2}H_{9}SN_{5}O_{2}^{-}$ |
| $65.01002$ | $55.23544$ | $SiH_{5}O_{2}^{-}$ | $168.040352$ | $-14.6108$ | $C_{8}H_{8}O_{4}^{-}$ |
| $67.021901$ | $44.213327$ | $C_{4}H_{3}O^{-}$ | $169.048167$ | $-14.5829$ | $C_{8}H_{9}O_{4}^{-}$ |
| $68.02015$ | $-15.612083$ | $C_{3}H_{5}Al^{-}$ | $174.038081$ | $-15.6003$ | $C_{6}H_{8}NO_{5}^{-}$ |
| $69.008199$ | $-17.932738$ | $C_{2}HN_{2}O^{-}$ | $175.025597$ | $4.487454$ | $C_{6}H_{7}O_{6}^{-}$ |
| $70.030792$ | $13.628371$ | $C_{3}H_{4}NO^{-}$ | $176.03275$ | $0.646721$ | $C_{6}H_{8}O_{6}^{-}$ |
| $73.93028$ | $-7.188817$ | $NiO^{-}$ | $178.027604$ | $2.50867$ | $C_{9}H_{6}O_{4}^{-}$ |
| $74.014904$ | $-17.496821$ | $C_{6}H_{2}^{-}$ | $179.065704$ | $23.6327$ | $C_{12}H_{7}N_{2}^{-}$ |
| $74.950035$ | $12.844876$ | $AlSO^{-}$ | $180.063123$ | $-4.51937$ | $C_{6}H_{12}O_{6}^{-}$ |
| $74.999841$ | $-28.952137$ | $SiH_{3}N_{2}O^{-}$ | $181.076324$ | $10.39675$ | $C_{9}H_{11}NO_{3}^{-}$ |
| $76.00656$ | $-1.795744$ | $C_{4}N_{2}^{-}$ | $182.023362$ | $39.35797$ | $C_{15}H_{2}^{-}$ |
| $76.963726$ | $-12.940662$ | $AlH_{2}SO^{-}$ | $183.01373$ | $8.697417$ | $C_{8}H_{7}SO_{3}^{-}$ |
| $77.015324$ | $-13.341718$ | $C_{3}H_{6}Cl^{-}$ | $188.053137$ | $-10.4537$ | $C_{5}H_{8}N_{4}O_{4}^{-}$ |
| $77.968974$ | $-11.316544$ | $CH_{3}PS^{-}$ | $189.033504$ | $15.54456$ | $C_{9}H_{5}N_{2}O_{3}^{-}$ |
| $78.02633$ | $-4.636106$ | $CH_{7}O_{2}Al^{-}$ | $196.039714$ | $-47.1399$ | $C_{8}H_{8}N_{2}O_{4}^{-}$ |
| $78.965902$ | $-0.438415$ | $CSOF^{-}$ | $197.023756$ | $8.668517$ | $C_{5}H_{10}PO_{6}^{-}$ |
| $79.962448$ | $3.925204$ | $TiNOH_{2}^{-}$ | $200.021559$ | $-14.1725$ | $C_{8}H_{9}PO_{4}^{-}$ |
| $80.045247$ | $11.76319$ | $C_{3}H_{6}F_{2}^{-}$ | $203.065952$ | $-26.6696$ | $C_{12}H_{11}O_{3}^{-}$ |
| $80.972312$ | $-18.480292$ | $AlOF_{2}^{-}$ | $205.041436$ | $-44.8512$ | $C_{11}H_{9}O_{4}^{-}$ |
| $81.03511$ | $6.43491$ | $C_{5}H_{5}O^{-}$ | $207.028611$ | $0.272883$ | $C_{8}H_{5}N_{3}O_{4}^{-}$ |
| $81.978802$ | $-15.682338$ | $C_{3}Na_{2}^{-}$ | $211.049267$ | $-28.6979$ | $C_{17}H_{7}^{-}$ |
| $82.033087$ | $-46.021116$ | $C_{4}H_{7}Al^{-}$ | $212.045506$ | $-23.8935$ | $C_{16}H_{6}N^{-}$ |
| $82.9656$ | $3.605366$ | $Si_{2}CHN^{-}$ | $213.0407$ | $1.119073$ | $C_{9}H_{9}O_{6}^{-}$ |
| $83.027506$ | $-33.073115$ | $C_{5}H_{4}F^{-}$ | $219.143058$ | $18.27564$ | $C_{14}H_{19}O_{2}^{-}$ |
| $83.969028$ | $-10.973804$ | $P_{2}H_{3}F^{-}$ | $223.037741$ | $-10.4311$ | $C_{14}H_{7}O_{3}^{-}$ |
| $84.967617$ | $-8.913735$ | $Si_{2}HN_{2}^{-}$ | $225.063843$ | $-31.6838$ | $C_{18}H_{9}^{-}$ |
| $85.031037$ | $18.038165$ | $C_{4}H_{5}O_{2}^{-}$ | $227.081442$ | $-22.8171$ | $C_{18}H_{11}^{-}$ |
| $85.96174$ | $-27.987373$ | $C_{3}H_{2}Ti^{-}$ | $235.103527$ | $13.88615$ | $C_{16}H_{13}NO^{-}$ |
| $86.023714$ | $-12.071431$ | $C_{3}H_{4}NO_{2}^{-}$ | $237.091499$ | $-2.54687$ | $C_{16}H_{13}O_{2}^{-}$ |
| $87.015088$ | $3.102877$ | $C_{3}H_{5}SN^{-}$ | $241.116789$ | $35.84011$ | $C_{12}H_{17}O_{5}^{-}$ |
| $88.03908$ | $-15.010835$ | $C_{3}H_{6}NO_{2}^{-}$ | $247.180743$ | $42.03538$ | $C_{16}H_{23}O_{2}^{-}$ |
| $89.01515$ | $-13.494921$ | $C_{4}H_{6}Cl^{-}$ | $250.051714$ | $-47.3029$ | $C_{16}H_{10}O_{3}^{-}$ |
| $90.005635$ | $0.652453$ | $C_{4}H_{5}^{37}Cl^{-}$ | $253.109924$ | $30.22327$ | $C_{20}H_{13}^{-}$ |
| $90.996736$ | $-2.105811$ | $SiH_{3}N_{2}O_{2}^{-}$ | $257.118004$ | $-60.5538$ | $C_{20}H_{17}^{-}$ |
| $93.035074$ | $-7.06264$ | $C_{3}H_{6}O_{2}F^{-}$ | $260.898995$ | $25.47971$ | $Na_{3}P_{2}H_{2}O_{8}^{-}$ |
| $93.983316$ | $-9.90675$ | $SN_{2}O_{2}H_{2}^{-}$ | $262.202461$ | $32.92194$ | $C_{17}H_{26}O_{2}^{-}$ |
| $94.030234$ | $4.223805$ | $C_{5}H_{4}NO^{-}$ | $265.133291$ | $15.13987$ | $C_{11}H_{21}O_{7}^{-}$ |
| $95.024035$ | $-11.062703$ | $C_{4}H_{3}N_{2}O^{-}$ | $267.111422$ | $-15.4719$ | $C_{14}H_{20}PO_{3}^{-}$ |
| $96.020489$ | $1.603921$ | $C_{3}H_{2}N_{3}O^{-}$ | $269.159976$ | $-58.9077$ | $C_{15}H_{25}O_{4}^{-}$ |
| $98.01688$ | $-4.709552$ | $C_{5}H_{3}OF^{-}$ | $275.145907$ | $-14.9194$ | $C_{13}H_{23}O_{6}^{-}$ |
| $99.008576$ | $-1.932188$ | $C_{4}H_{3}O_{3}^{-}$ | $279.157468$ | $-9.72358$ | $C_{16}H_{23}O_{4}^{-}$ |
| $100.039204$ | $-11.972486$ | $C_{4}H_{6}NO_{2}^{-}$ | $281.159275$ | $-4.6302$ | $C_{12}H_{25}O_{7}^{-}$ |
| $101.03343$ | $-21.984627$ | $C_{3}H_{5}N_{2}O_{2}^{-}$ | $283.189464$ | $-7.13026$ | $C_{16}H_{27}O_{4}^{-}$ |
| $104.034135$ | $-11.362073$ | $C_{3}H_{6}NO_{3}^{-}$ | $293.165508$ | $7.677389$ | $C_{16}H_{23}NO_{4}^{-}$ |
| $106.004612$ | $-0.692149$ | $C_{4}N_{3}O^{-}$ | $297.162494$ | $-8.01052$ | $C_{23}H_{21}^{-}$ |
| $106.990024$ | $-3.082792$ | $C_{2}H_{4}PO_{3}^{-}$ | $298.167588$ | $-17.1421$ | $C_{23}H_{22}^{-}$ |
| $107.983086$ | $-20.436657$ | $C_{5}O_{3}^{-}$ | $299.146436$ | $-11.9557$ | $C_{15}H_{23}O_{6}^{-}$ |
| $108.047085$ | $-3.980552$ | $CH_{8}SN_{4}^{-}$ | $301.139806$ | $-6.77815$ | $C_{14}H_{17}N_{6}O_{2}^{-}$ |
| $108.901634$ | $-2.999333$ | $KCl_{2}^{-}$ | $309.16312$ | $-5.67449$ | $C_{24}H_{21}^{-}$ |
| $109.02024$ | $-0.530903$ | $C_{2}H_{7}SNO_{2}^{-}$ | $311.18406$ | $11.36153$ | $C\_24H\_23-$ |
| $110.022126$ | $-11.663116$ | $C_{3}H_{2}N_{4}O^{-}$ | $313.152666$ | $-15.1799$ | $C_{16}H_{26}PO_{4}^{-}$ |
| $112.036912$ | $-19.166549$ | $C_{3}H_{4}N_{4}O^{-}$ | $325.1968$ | $1.922692$ | $C_{25}H_{25}^{-}$ |
| $115.939829$ | $1.934539$ | $C_{3}S_{2}O^{-}$ | $326.202245$ | $-23.3837$ | $C_{18}H_{30}O_{5}^{-}$ |
| $116.070762$ | $-8.096272$ | $C_{5}H_{10}NO_{2}^{-}$ | $333.145835$ | $59.18371$ | $C_{18}H_{22}PO_{4}^{-}$ |
| $116.996078$ | $-7.030702$ | $C_{2}HOF_{4}^{-}$ | $339.216824$ | $49.77771$ | $C_{19}H_{31}SO_{3}^{-}$ |
| Footnotes:  *m/z*^–^_obs._: observed mass to charge ratio in the negative ion mode.  ΔM: ΔM = 10^6^ × (*m/z*^–^_obs._− *m/z*^–^_theo_.)/ *m/z*^–^_theo._ (expressed in ppm) [9, 10].  Peak identifications using the IONTOF SurfaceLab software | | | | | |

**Table S6:** ToF-SIMS spectral peaks observed for TSB media in the positive mode.

| *m/z*^−^*_obs._* | ∆M, ppm | Species | *m/z*^−^*_obs._* | ∆M, ppm | Species |
| --- | --- | --- | --- | --- | --- |
| $1.007328$ | $50.996324$ | $H^{+}$ | $85.991046$ | $-8.525308$ | $C_{4}H_{3}Cl^{+}$ |
| $2.015136$ | $16.990899$ | $H_{2}^{+}$ | $86.096531$ | $1.216398$ | $C_{5}H_{12}N^{+}$ |
| $3.022966$ | $13.194826$ | $H_{3}^{+}$ | $86.955468$ | $-24.379744$ | $CH_{3}SCa^{+}$ |
| $11.999681$ | $19.15662$ | $C^{+}$ | $86.984656$ | $5.983876$ | $C_{4}ONa^{+}$ |
| $13.007553$ | $21.254061$ | $CH^{+}$ | $87.09316$ | $17.053017$ | $C_{4}H_{11}N_{2}^{+}$ |
| $14.015025$ | $-5.444556$ | $CH_{2}^{+}$ | $89.0383$ | $-3.105128$ | $C_{7}H_{5}^{+}$ |
| $15.022989$ | $4.15857$ | $CH_{3}^{+}$ | $90.974291$ | $-25.87037$ | $CHO_{2}Na_{2}^{+}$ |
| $17.026197$ | $11.560183$ | $NH_{3}^{+}$ | $91.049564$ | $-7.029416$ | $C_{2}H_{7}N_{2}O_{2}^{+}$ |
| $19.017935$ | $4.939213$ | $H_{3}O^{+}$ | $94.027244$ | $-1.636216$ | $C_{3}H_{2}N_{4}^{+}$ |
| $27.022939$ | $0.455896$ | $C_{2}H_{3}^{+}$ | $96.07729$ | $-36.280805$ | $C_{6}H_{10}N^{+}$ |
| $28.019064$ | $31.700956$ | $CH_{2}N^{+}$ | $96.921446$ | $-3.452932$ | $KNaCl^{+}$ |
| $29.001742$ | $-15.472568$ | $CHO^{+}$ | $97.008307$ | $-1.1592$ | $C_{5}H_{2}OF^{+}$ |
| $29.025674$ | $-11.240408$ | $CH_{3}N^{+}$ | $98.060209$ | $1.718283$ | $C_{5}H_{8}NO^{+}$ |
| $29.038703$ | $4.365588$ | $C_{2}H_{5}^{+}$ | $98.917015$ | $-60.873373$ | $Ca_{2}F^{+}$ |
| $31.01752$ | $-10.368114$ | $CH_{3}O^{+}$ | $100.084838$ | $-20.838281$ | $C_{4}H_{10}N_{3}^{+}$ |
| $32.049386$ | $-2.804326$ | $CH_{6}N^{+}$ | $102.046866$ | $-6.65189$ | $C_{5}H_{7}OF^{+}$ |
| $37.005025$ | $4.169465$ | $CH_{2}Na^{+}$ | $103.04951$ | $-6.732042$ | $C_{3}H_{7}N_{2}O_{2}^{+}$ |
| $38.017098$ | $52.529246$ | $C_{3}H_{2}^{+}$ | $104.10715$ | $1.530351$ | $C_{5}H_{14}NO^{+}$ |
| $40.994341$ | $-56.064836$ | $CH_{2}Al^{+}$ | $104.9915$ | $-0.515303$ | $C_{5}H_{2}OAl^{+}$ |
| $42.035236$ | $33.554671$ | $C_{2}H_{4}N^{+}$ | $105.063381$ | $-23.535338$ | $C_{3}H_{9}N_{2}O_{2}^{+}$ |
| $43.042837$ | $27.566769$ | $C_{2}H_{5}N^{+}$ | $106.94926$ | $-1.366795$ | $CHS_{2}NO^{+}$ |
| $44.021498$ | $31.406119$ | $CH_{5}Al^{+}$ | $107.044365$ | $-7.038693$ | $C_{2}H_{7}N_{2}O_{3}^{+}$ |
| $44.977498$ | $-36.0005$ | $SiHO^{+}$ | $108.941166$ | $-0.741006$ | $CHS_{2}O_{2}^{+}$ |
| $45.032911$ | $-12.885294$ | $C_{2}H_{5}O^{+}$ | $111.973743$ | $10.345745$ | $C_{3}SN_{2}O^{+}$ |
| $45.978938$ | $-1.157084$ | $Na_{2}^{+}$ | $112.035893$ | $-18.468454$ | $C_{3}H_{4}N_{4}O^{+}$ |
| $46.027903$ | $-18.180783$ | $CH_{4}NO^{+}$ | $116.068861$ | $-3.460545$ | $C_{3}H_{8}N_{4}O^{+}$ |
| $46.063599$ | $-33.13788$ | $C_{2}H_{8}N^{+}$ | $116.892206$ | $13.975826$ | $K_{3}^{+}$ |
| $46.98808$ | $-24.293892$ | $C_{2}Na^{+}$ | $117.00032$ | $-2.875149$ | $Si_{3}C_{2}H_{9}^{+}$ |
| $50.983675$ | $-9.038449$ | $CONa^{+}$ | $117.99422$ | $-1.383098$ | $CH_{2}SN_{4}O^{+}$ |
| $51.020381$ | $-49.889518$ | $C_{4}H_{3}^{+}$ | $118.087253$ | $8.452129$ | $C_{5}H_{12}NO_{2}^{+}$ |
| $52.014482$ | $-24.774425$ | $CH_{3}NNa^{+}$ | $120.004764$ | $-4.674745$ | $C_{3}H_{4}O_{5}^{+}$ |
| $52.027795$ | $-56.823199$ | $C_{4}H_{4}^{+}$ | $120.078633$ | $4.474213$ | $C_{5}H_{12}O_{3}^{+}$ |
| $53.000798$ | $-26.280724$ | $C_{3}HO^{+}$ | $121.922229$ | $-3.224187$ | $CNOCa_{2}^{+}$ |
| $53.036583$ | $-37.595828$ | $C_{4}H_{5}^{+}$ | $124.937436$ | $-0.485225$ | $Na_{2}PO_{3}^{+}$ |
| $54.033601$ | $-4.154551$ | $C_{3}H_{4}N^{+}$ | $128.007232$ | $-5.732267$ | $C_{3}H_{3}O_{2}F_{3}^{+}$ |
| $55.016818$ | $-18.602732$ | $C_{3}H_{3}O^{+}$ | $129.939489$ | $-9.218384$ | $CNNa_{3}Cl^{+}$ |
| $55.052654$ | $-28.568052$ | $C_{4}H_{7}^{+}$ | $130.056117$ | $1.508724$ | $C_{5}H_{10}SN_{2}^{+}$ |
| $56.01712$ | $-53.458511$ | $C_{2}H_{5}Al^{+}$ | $130.085037$ | $0.960182$ | $C_{4}H_{10}N_{4}O^{+}$ |
| $56.04858$ | $-15.973842$ | $C_{3}H_{6}N^{+}$ | $131.0929$ | $1.237041$ | $C_{4}H_{11}N_{4}O^{+}$ |
| $57.016897$ | $24.455704$ | $SiC_{2}H_{5}^{+}$ | $131.936732$ | $-6.209318$ | $CH_{2}O_{3}Cl_{2}^{+}$ |
| $57.031597$ | $-33.213003$ | $C_{3}H_{5}O^{+}$ | $132.105613$ | $28.067125$ | $C_{6}H_{14}NO_{2}^{+}$ |
| $57.068396$ | $-25.949164$ | $C_{4}H_{9}^{+}$ | $133.09986$ | $-9.89414$ | $C_{10}H_{13}^{+}$ |
| $58.029989$ | $21.513381$ | $C_{2}H_{4}NO^{+}$ | $140.067777$ | $-10.604774$ | $C_{5}H_{8}N_{4}O^{+}$ |
| $58.064226$ | $-15.48694$ | $C_{3}H_{8}N^{+}$ | $140.904226$ | $-8.898405$ | $P_{3}O_{3}^{+}$ |
| $58.965098$ | $-13.573828$ | $NaHCl^{+}$ | $147.107808$ | $1.264638$ | $C_{7}H_{17}SN^{+}$ |
| $59.048404$ | $-12.484363$ | $C_{3}H_{7}O^{+}$ | $148.048816$ | $-4.192141$ | $C_{7}H_{7}F_{3}^{+}$ |
| $59.073867$ | $15.509925$ | $C_{3}H_{9}N^{+}$ | $150.051478$ | $33.83516$ | $C_{12}H_{6}^{+}$ |
| $60.053877$ | $-29.091398$ | $CH_{6}N_{3}^{+}$ | $151.057137$ | $-19.613538$ | $C_{5}H_{11}O_{5}^{+}$ |
| $60.081848$ | $17.850327$ | $C_{3}H_{10}N^{+}$ | $152.053692$ | $-28.520934$ | $C_{7}H_{8}N_{2}O_{2}^{+}$ |
| $61.008674$ | $4.169139$ | $C_{2}H_{2}OF^{+}$ | $154.077993$ | $1.888676$ | $C_{12}H_{10}^{+}$ |
| $61.951635$ | $-20.876558$ | $KNa^{+}$ | $154.873828$ | $35.959456$ | $P_{5}^{+}$ |
| $61.996527$ | $4.336335$ | $C_{2}F_{2}^{+}$ | $155.079343$ | $1.57532$ | $C_{5}H_{12}N_{2}O_{2}Na^{+}$ |
| $63.020181$ | $-43.567309$ | $C_{5}H_{3}^{+}$ | $156.03461$ | $-29.832974$ | $C_{5}H_{7}O_{2}F_{3}^{+}$ |
| $65.035574$ | $-46.169447$ | $C_{5}H_{5}^{+}$ | $160.029551$ | $-6.508374$ | $C_{5}H_{5}F_{5}^{+}$ |
| $65.996055$ | $-20.985699$ | $C_{3}NO^{+}$ | $161.874948$ | $32.236003$ | $Fe_{2}H_{2}O_{3}^{+}$ |
| $66.955971$ | $-31.393902$ | $COK^{+}$ | $162.043237$ | $-19.52915$ | $C_{13}H_{6}^{+}$ |
| $66.97827$ | $-11.652278$ | $CO_{2}Na^{+}$ | $166.075805$ | $-11.419416$ | $C_{13}H_{10}^{+}$ |
| $67.016063$ | $-6.497541$ | $C_{2}HN_{3}^{+}$ | $167.080051$ | $-32.774274$ | $C_{13}H_{11}^{+}$ |
| $67.05173$ | $-37.228465$ | $C_{5}H_{7}^{+}$ | $169.087477$ | $9.201678$ | $C_{9}H_{13}O_{3}^{+}$ |
| $68.017367$ | $-40.394015$ | $C_{3}H_{5}Al^{+}$ | $170.049058$ | $-34.447097$ | $C_{6}H_{9}O_{2}F_{3}^{+}$ |
| $68.04888$ | $-8.745582$ | $C_{4}H_{6}N^{+}$ | $175.110651$ | $-6.228917$ | $C_{12}H_{15}O^{+}$ |
| $69.03162$ | $-7.654964$ | $C_{2}H_{3}N_{3}^{+}$ | $176.055528$ | $-37.054554$ | $C_{14}H_{8}^{+}$ |
| $69.06726$ | $-37.879368$ | $C_{5}H_{9}^{+}$ | $177.012715$ | $-3.560727$ | $C_{5}H_{3}F_{6}^{+}$ |
| $70.026945$ | $-25.638947$ | $C_{3}H_{4}NO^{+}$ | $177.057104$ | $14.027529$ | $C_{10}H_{9}O_{3}^{+}$ |
| $70.068292$ | $45.194824$ | $C_{4}H_{8}N^{+}$ | $180.896697$ | $-5.245924$ | $Na_{3}S_{2}O_{3}^{+}$ |
| $71.984633$ | $35.674551$ | $CNNa_{2}^{+}$ | $182.071018$ | $-8.776424$ | $C_{13}H_{10}O^{+}$ |
| $72.077433$ | $-46.380683$ | $C_{4}H_{10}N^{+}$ | $186.904721$ | $-38.678866$ | $Na_{4}PO_{4}^{+}$ |
| $72.983198$ | $-4.229608$ | $CaNOH_{3}^{+}$ | $188.056247$ | $11.913194$ | $C_{5}H_{8}N_{4}O_{4}^{+}$ |
| $73.024389$ | $-21.522619$ | $CH_{4}F_{3}^{+}$ | $191.072511$ | $-2.302523$ | $C_{14}H_{9}N^{+}$ |
| $73.060923$ | $23.027292$ | $C_{3}H_{10}Al^{+}$ | $192.028992$ | $-45.28206$ | $C_{5}H_{8}N_{2}O_{6}^{+}$ |
| $74.02125$ | $-32.489685$ | $C_{2}H_{4}NO_{2}^{+}$ | $194.010809$ | $-18.763641$ | $C_{8}H_{6}SN_{2}O_{2}^{+}$ |
| $74.059891$ | $-2.018492$ | $C_{3}H_{8}NO^{+}$ | $196.868234$ | $-0.662536$ | $K_{2}NaSO_{4}^{+}$ |
| $74.096405$ | $-0.285185$ | $C_{4}H_{12}N^{+}$ | $197.09038$ | $-28.977439$ | $C_{14}H_{13}O^{+}$ |
| $75.027823$ | $20.340077$ | $C_{3}H_{7}S^{+}$ | $202.877069$ | $-43.465576$ | $KNa_{3}PO_{4}^{+}$ |
| $77.03393$ | $-60.311234$ | $C_{6}H_{5}^{+}$ | $205.086113$ | $49.190968$ | $C_{14}H_{9}N_{2}^{+}$ |
| $77.92312$ | $-48.058375$ | $K_{2}^{+}$ | $213.062059$ | $5.424638$ | $C_{5}H_{17}O_{5}Si_{2}^{+}$ |
| $78.954937$ | $-9.254801$ | $KNaOH^{+}$ | $219.075271$ | $-23.598855$ | $C_{16}H_{11}O^{+}$ |
| $80.047125$ | $-29.36995$ | $C_{5}H_{6}N^{+}$ | $221.136332$ | $-21.247581$ | $C_{13}H_{19}NO_{2}^{+}$ |
| $80.948651$ | $-33.106723$ | $KNaF^{+}$ | $249.165187$ | $5.659948$ | $C_{19}H_{21}^{+}$ |
| $82.060042$ | $-61.94987$ | $C_{5}H_{8}N^{+}$ | $263.180751$ | $5.029469$ | $C_{20}H_{23}^{+}$ |
| $83.047051$ | $-9.000612$ | $C_{3}H_{5}N_{3}^{+}$ | $264.181387$ | $25.404877$ | $C_{19}H_{22}N^{+}$ |
| $84.043414$ | $-11.61778$ | $C_{4}H_{6}NO^{+}$ | $271.145213$ | $-23.53675$ | $C_{12}H_{24}O_{4}ONa^{+}$ |
| $84.07932$ | $-17.314349$ | $C_{5}H_{10}N^{+}$ | $281.13395$ | $5.237992$ | $C_{22}H_{17}^{+}$ |
| $84.991285$ | $-8.652532$ | $C_{3}HO_{3}^{+}$ | $285.158717$ | $35.755563$ | $C_{18}H_{21}O_{3}^{+}$ |
| $85.083399$ | $-61.136979$ | $C_{5}H_{11}N^{+}$ | $297.15999$ | $-12.745211$ | $C_{23}H_{21}^{+}$ |
| Footnotes:  *m/z*^–^_obs._: observed mass to charge ratio in the negative ion mode.  ΔM: ΔM = 10^6^ × (*m/z*^–^_obs._− *m/z*^–^_theo_.)/ *m/z*^–^_theo._ (expressed in ppm) [9, 10].  Peaks identifications using the IONTOF SurfaceLab software | | | | | |
